# Acute Illness and Death in Children With Adrenal Insufficiency

**DOI:** 10.3389/fendo.2021.757566

**Published:** 2021-10-13

**Authors:** Chris Worth, Avni Vyas, Indraneel Banerjee, Wei Lin, Julie Jones, Helen Stokes, Nicci Komlosy, Steven Ball, Peter Clayton

**Affiliations:** ^1^ Department of Paediatric Endocrinology, Royal Manchester Children’s Hospital, Manchester, United Kingdom; ^2^ School of Medicine, Faculty of Biology, Medicine and Health, University of Manchester, Manchester, United Kingdom; ^3^ Department of Endocrinology, Manchester Royal Infirmary, Manchester, United Kingdom

**Keywords:** adrenal insufficiency, adrenal crisis, hydrocortisone, outcomes, socioeconomic

## Abstract

**Background:**

Adrenal Insufficiency (AI) can lead to life-threatening Adrenal Crisis (AC) and Adrenal Death (AD). Parents are trained to prevent, recognise and react to AC but there is little available information on what parents are actually doing at home to manage symptomatic AI.

**Methods:**

Three approaches were taken: (A) A retrospective analysis of patient characteristics in children and young people with AD over a 13-year period, (B) An interview-aided questionnaire to assess the circumstances around AC in children currently in our adrenal clinic, and (C) a separate study of parent perceptions of the administration of parenteral hydrocortisone.

**Results:**

Thirteen patients died (median age 10 years) over a thirteen-year period resulting in an estimated incidence of one AD per 300 patient years. Those with unspecified adrenal insufficiency were overrepresented (*P* = 0.004).

Of the 127 patients contacted, thirty-eight (30%) were identified with hospital attendance with AC. Responses from twenty patients (median age 7.5 years) with AC reported nausea/vomiting (75%) and drowsiness (70%) as common symptoms preceding AC. All patients received an increase in oral hydrocortisone prior to admission but only two received intramuscular hydrocortisone.

Questionnaires revealed that 79% of parents reported confidence in the administration of intramuscular hydrocortisone and only 20% identified a missed opportunity for injection.

**Conclusions:**

In children experiencing AC, parents followed ‘sick day’ guidance for oral hydrocortisone, but rarely administered intramuscular hydrocortisone. This finding is discrepant from the 79% of parents who reported confidence in this task. Local training programmes for management of AC are comprehensive, but insufficient to prevent the most serious crises. New strategies to encourage use of parenteral hydrocortisone need to be devised.

## 1 Introduction

Adrenal insufficiency (AI) can be due to several conditions presenting in children and young people. Congenital adrenal hyperplasia (CAH), comprises the majority of AI in children with smaller contributions from autoimmune, adrenal dysgenesis and central causes (1). The unifying feature is the requirement for glucocorticoid replacement therapy and the subsequent risk of adrenal crisis (AC) if doses are suboptimal. There is no universal definition of AC for children as presentation is variable and a pragmatic diagnosis of AC is based an acute deterioration in health with haemodynamic disturbance (2).

Risk of AC in patients with AI is variable but, in children with CAH, up to 70% of ACs occur in the first 10 years of life (3) with a rate of 6.5-10.9 crises/100 patient years (4, 5). When all causes of AI are considered, the rate of AC is 3.4 crises/100 patient years in children (6). The only satisfactory predictor of AC is a previous AC (7). Risk of adrenal death (AD) is significant with Standardised Mortality Ratio (SMR) at 9.6-17.4 (8, 9) and 1 death per 205 patients years (9) in children with AI. Rates of both AD and AC are likely to be higher than the literature suggests with recent studies reporting a median of zero AC events per patient year (10).

There is little evidence about background risk factors such as ethnicity and socioeconomic status on risk of AC and AD. One study reported a higher SMR (20.4 *vs* 1.1) in those of Indian ethnicity than white British ethnicity in UK children (11). No studies have investigated the association of deprivation indices with AC or AD.

Multiple studies have reported symptoms of children with AC as interpreted (4) or documented by medical staff (12). There are no cohort studies in children directly examining parent reported symptoms in the period prior to an admission with acute illness.

Our overall aim was to better understand the incidence of AD and the events leading up to this. We thus undertook three separate, but related studies. We investigated the incidence and risk factors for AD. Secondly, we aimed to investigate pre-hospital symptomatic adrenal insufficiency and adrenal crisis as well as parental management in order to better understand events leading up to hospital admission. Finally, we undertook an investigation into the perceptions of and reasons behind parental actions at times of AC.

## 2 Methods

### 2.1 Part A – Incidence of and Risk Factors for AD

The study team retrospectively reviewed medical notes and coroner’s reports for likely cause of death in all patients with AI under 25 years of age who died between 2006 and 2019 and excluded those who had died from causes unrelated to AI such as malignancies or trauma. This cohort of patients included all patients under the age of 25 years of age who were under the care of the endocrinology teams at Royal Manchester Children’s Hospital and Manchester Royal Infirmary (both Manchester Foundation Trust). Patients may have died at any hospital or out of hospital and all deaths were brought to the attention of the endocrinology teams. If it was not possible to ascertain cause of death from medical notes adrenal insufficiency was assumed as a contributing factor. It was decided not to contact parents and families for more information to avoid unnecessary distress. IMD scores in patients with AD were compared with a list of patients with AI but no AD provided by the hospital coding department at Manchester Foundation Trust. These patients had a coded diagnosis of AI as identified by the coding department but had not died. They were utilised for comparing demographics with those patients who had AD, as hospital electronic records were available for these patients for verification. It was acknowledged that this group represented a cross sectional number of patients but did not represent the full cohort of patients with AI under the care of the endocrinology teams. Thus this list of patients was not suitable as a denominator for calculation of the rate of AD. A more realistic AI denominator for AD was provided by numbers of patients registered in paediatric adrenal and endocrine clinics and young adult endocrine clinics. This number is an average for the 13-year study period and thus the rate of AD is reported as an estimate due to a small degree of uncertainty about the exact value of the denominator.

### 2.2 Part B - Questionnaires on Acute Illness

Over a six-month period, specialist nurses approached patients in the North West paediatric endocrine clinic. All those with previous Emergency Department attendances with acute illness between March 2011 and 2019 were eligible for inclusion including those for whom this was the initial presentation of AI. All patients were under the care of the specialist endocrine team but attended multiple Emergency Departments depending on their proximity to local hospitals. Questionnaires were administered in adrenal clinic with three follow up telephone reminders over the subsequent 16 weeks. Questionnaires were retrospective and asked parents to state whether their child had exhibited any of the following symptoms: vomiting, tummy pain, confusion, unable to wake, slurred speech, severe tiredness, high temperature, very sleepy, not responsive and convulsions/fits. The original questionnaire is provided as an appendix. Additional demographic data were derived from hospital electronic patient records.

AC was defined as any acute illness requiring attendance at the emergency department accompanied by parenteral hydrocortisone administration and reported reduced level of consciousness indicating haemodynamic compromise and/or health professional documentation of AC. Diagnosis of AC in retrospect is challenging and our definition fits that provided by Rushworth et al. (2).

Socioeconomic status was based on Index of Multiple Deprivation (IMD) scores [http://imd-by-geo.opendatacommunities.org/imd/2019/area] calculated from postcodes, representing a rank from 1 (most deprived) to 32,844 (least deprived). These scores were compared with a list of patients under 18 years of age with AI without AC over the same period provided by hospital coding.

### 2.3 Part C - Parental Perceptions of Parenteral Hydrocortisone

A further study was conducted between January and February 2020 on a separate group of patients identified in the same clinic and drawn from the same population as Part B. We investigated the perceptions of the parents on the use of intramuscular hydrocortisone. Questionnaires were completed in clinic and addressed whether parents had hydrocortisone emergency kits at home, knew how to administer them, whether they had ever used them and if opportunities to inject had been missed.

### 2.4 Statistical Analysis

Descriptive statistical methods were analysed using SPSS 25 (IBM). Non-parametric tests were used to assess for difference between groups as data were not normally distributed. Logistic regression was used to adjust for confounding factors.

As AC and AD are rare events and the design was that of a service evaluation, the sample size was not powered for any predetermined outcome and formal ethical approval was not required.

As the project was conducted as a quality improvement project to evaluate the performance of our clinical service patient consent and ethical approval was not required.

## 3 Results

### 3.1 Part A - Adrenal Deaths

During the study period thirteen patients (under the age of 25 years) were identified with AD. Seven (54%) were female and most were of European ethnicity (**Table 1**). Unspecified adrenal insufficiency was the most common diagnosis (cause either not ascertained or not investigated prior to AD but patient known to have AI and treated with steroid replacement) with hypopituitarism being the most common specific diagnosis (**Table 1**). Mean age at death was 10 years (range 3 months to 24 years).

**Table 1:** Demographics of patients with AD.

Characteristic	AI with AD n = 13 (%)	AI without AD n = 107 (%)	*P*
Sex			0.415
Male	6 (46)	56 (52)	
Female	7 (54)	51 (48)	
Ethnicity			0.342
European	7 (54)	62 (58)	
South Asian	2 (15)	29 (27)	
African Caribbean	1 (8)	6 (5)	
Mixed Ethnicity	1 (8)	2 (2)	
Other	0 (0)	4 (4)	
Unknown	2 (15)	4 (4)	
Diagnosis			0.004*
CAH	3 (23)	45 (42)	
Hypopituitarism	2 (15)	49 (46)	
Secondary to steroid Rx	1 (8)	3 (3)	
Addison’s/auto-immune	0	5 (5)	
Unspecified	5 (38)	4 (4)	
Other	2 (8)	1 (1)	

CAH, Congenital Adrenal Hyperplasia; P, P value for difference between groups calculated via Logistic regression. Nine patients did not have a specific diagnosis for the cause of their adrenal insufficiency.

*Results demonstrate a significant difference in underlying diagnoses between those with and without AD: CAH and Hypopituitarism are relatively underrepresented in the group with AD perhaps suggesting that those with an unclear diagnosis for their AI are at higher risk.

Initial demographic comparisons (with a list of 107 AI patients without AD provided by the hospital coding department) showed a relative overrepresentation of unspecified AI in the AD group with both CAH and hypopituitarism relatively underrepresented (Chi-Square = 33.32, *P <*0.001) (**Table 1**). This significance persisted when adjusting for confounding factors (IMD score, gender, ethnicity) in logistic regression analysis (*P* = 0.004). There was no difference between groups with and without AD with respect to IMD score (*P* = 0.648) or ethnicity (*P* = 0.342). There was no significant difference in rate of AD to no AD between those with primary and secondary AI (3/53 (6%) *vs* 10/57 (17%), Chi-square = 2.63, *P* = 0.1).

The AI group used for demographic comparisons was not suitable to be used as a denominator for AD rate, as it did not represent the whole population. Over the period of the study there were an estimated 300 children and young people with AI actively managed under the care of endocrine services in Manchester, based on the average numbers registered in paediatric and young adult clinics throughout the study period of 13 years. Given 13 deaths over 13 years this amounts to one death per year and an estimated incidence of 1 death per 300 patient years in our cohort.

### 3.2 Part B - Emergency Department Attendances

**Figure 1:**
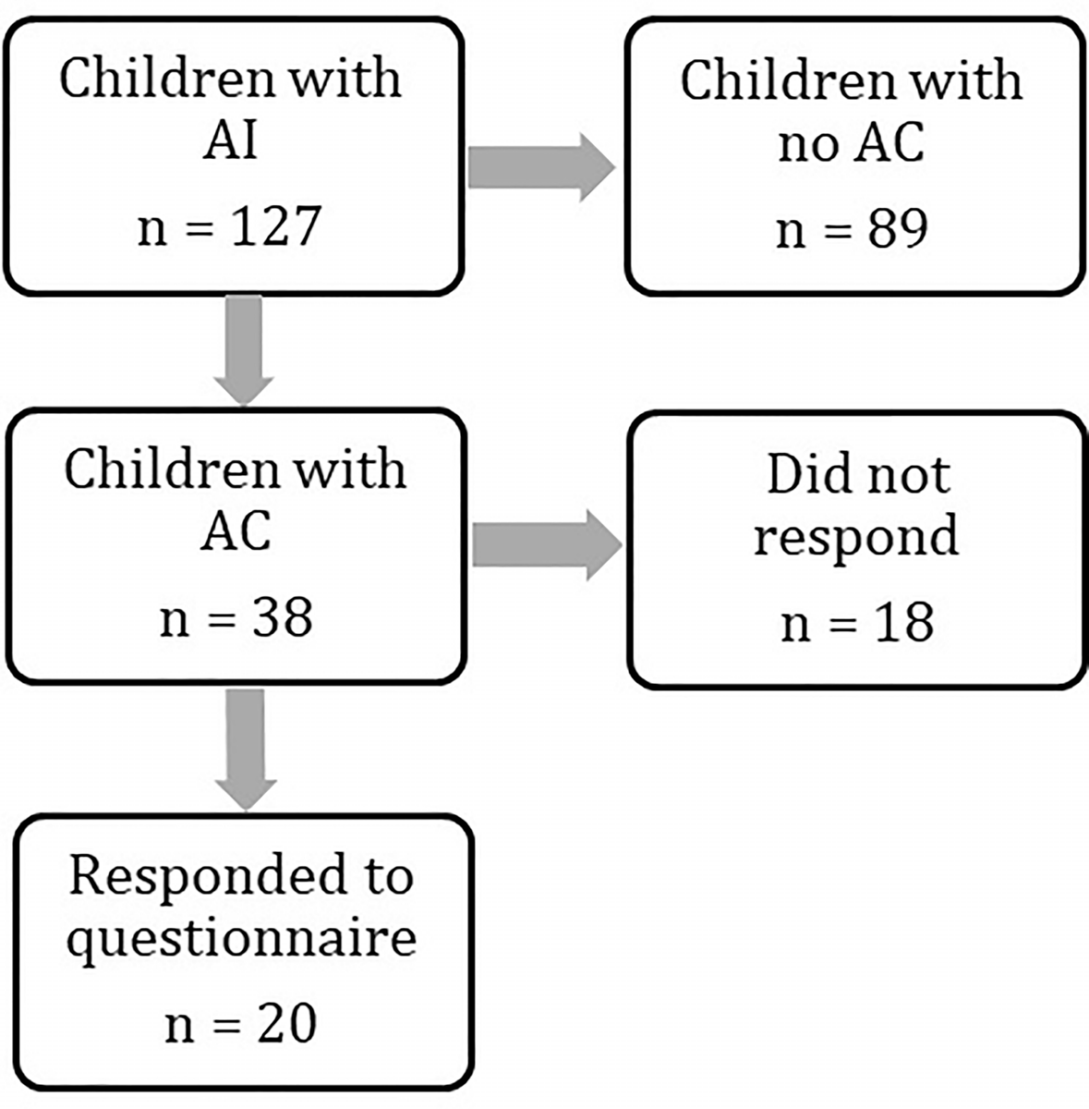
Flowchart demonstrating numbers of patients identified at each stage of the study. Patient groups were compared at every stage to assess for differences. Results are provided in the manuscript and **Tables 1** and **2**.

We identified 127 children with AI under the age of 18 years from the regional endocrine clinic. Of these 127 children, 38 (30%) had a recorded attendance at the Emergency Department with acute illness between 2011 and 2019. Twenty patients (52%) completed the questionnaire (**Figure 1**), and all satisfied our definition of AC. Two of these patients did not have a prior diagnosis of AI at the time of AC and thus questions regarding pre-hospital hydrocortisone treatment were not answered. Demographics are presented in **Table 2**. Mean age (range) at admission was 7.5 years (1.2 years - 17.6 years) with mean time from diagnosis of 6.5 years (10 days to 15 years).

**Table 2:** Demographics of AI patients with and without AC.

Characteristic	AI but no AC n = 89 (%)	AI with AC n = 38 (%)	P
Sex			0.50
Male	44 (49)	22 (58)	
Female	45 (51)	16 (42)	
Ethnicity			0.99
European	50 (56)	25 (66)	
South Asian	26 (29)	8 (21)	
African Caribbean	5 (6)	2 (5)	
Mixed ethnicity	2 (2)	1 (3)	
Not reported	3 (3)	1 (3)	
Other	3 (3)	1 (3)	
Diagnosis			0.95
CAH	41 (46)	17 (45)	
Unspecified	3 (3)	4 (11)	
Hypopituitarism	38 (43)	13 (34)	
Addison’s/auto-immune	4 (4)	2 (5)	
No prior diagnosis of AI	0 (0)	1 (3)	
Secondary to steroid Rx	2 (2)	1 (3)	
Other	1 (1)	0 (0)	
IMD	9797	14754	0.06

CAH = congenital adrenal hyperplasia. Rx = medication. IMD = Index of Multiple Deprivation Score. P = P value calculated via Logistic regression. There were no significant differences in gender, diagnosis, ethnicity or IMD score between groups with and without AC.

Demographics of patients with AC were compared with those AI patients without AC. No statistical differences were found in IMD score, gender, diagnosis or ethnicity between the two groups in logistic regression analysis nor individual Chi-square tests (**Table 2**). There was no difference in rate of AC to no AC between those with primary and secondary AI (19/63 (30%) *vs* 19/64 (30%), Chi-square = 0.003, *P* = 0.95). Demographics of patients with AC were compared between responders and non-responders to the questionnaire. There were no differences between groups in logistic regression analysis.

#### 3.2.1 Symptoms

**Figure 2:**
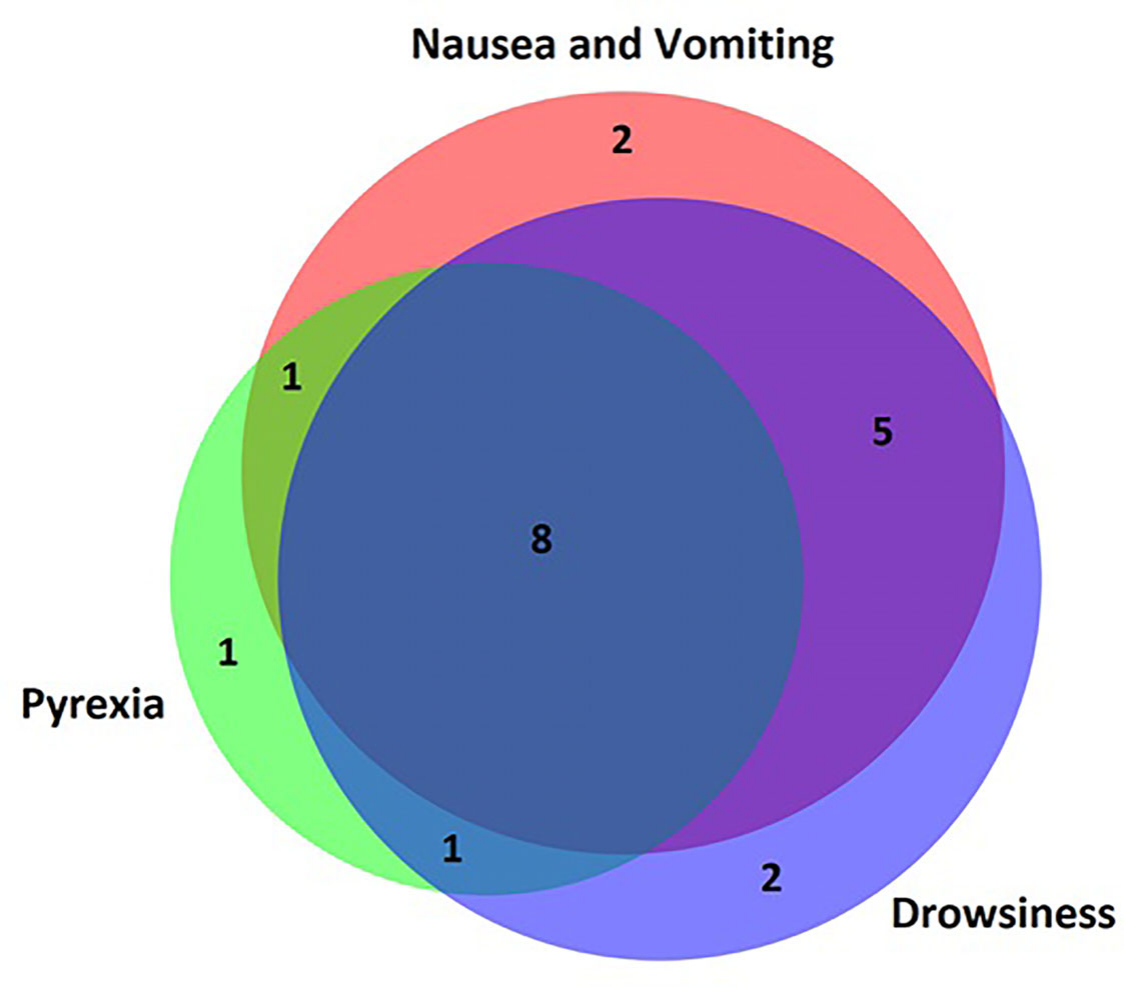
Venn diagram of parent-reported symptoms. Most patients presented with overlapping symptoms and very few patients presented to hospital with an isolated symptom. Nausea/vomiting, pyrexia and drowsiness were the most common parent-reported symptoms.

The most common symptom reported by patients’ parents prior to presentation to the emergency department was nausea and vomiting in 15 (75%) patients. Reduced conscious level was common and varied from drowsiness in 14 (70%), severe lethargy in 10 (50%) and being completely unrousable in 4 (20%) patients. Parentally reported pyrexia was present in 11 (55%) cases. Other symptoms were less common and are summarised in **Table 3**. One patient had none of the symptoms listed in the questionnaire and AC was diagnosed *via* observations performed by the primary care physician at a routine checkup. There was significant overlap between symptoms (**Figure 2**) with very few patients presenting with isolated symptoms.

**Table 3:** Symptoms of patients presenting to hospital with adrenal crisis.

Symptom	Number (%)
Conscious level	
Drowsiness	14 (70)
Severe Lethargy	10 (50)
Unrousable	4 (20)
Gastrointestinal	
Nausea and Vomiting	15 (75)
Abdo pain	5 (25)
Neurological	
Confusion	4 (20)
Incoherent speech	2 (10)
Convulsions	2 (10)
Pyrexia	11 (55%)

n = 20. Pyrexia = parental reported “high temperature”. Abdo pain = parental reported “tummy pain”. As detailed in the manuscript, the most commonly reported symptoms were nausea and vomiting, reduced conscious level and pyrexia.

#### 3.2.2 Hydrocortisone Dosing

All patients in our department are provided with individualised “sick day rules”. One patient’s parents did not answer questions about hydrocortisone dosing. Of the 17 patients with known AI (at the time of AC) who answered the question about dosing, 16 had their oral hydrocortisone dose doubled or tripled in the 48 hours prior to admission.

Of the 20 patients, two had no prior diagnosis of AI, and two patients had neither vomiting nor reduced level of consciousness and therefore did not require parenteral hydrocortisone at home. Of 16 patients who required parenteral hydrocortisone (as per specialist nurse instructions provided at diagnosis), only two received this from parents, leaving the rest to receive this from paramedics or emergency department staff.

### 3.3 Part C - Parental Perceptions of Emergency Hydrocortisone

Questionnaires investigating parental perceptions of emergency hydrocortisone for AC were distributed to a separate cohort of parents of 43 children with AI. Demographic details and diagnoses were not collected at this stage. The majority [41 (95%)] possessed an in-date emergency hydrocortisone kit at home but only 32 (74%) reported that the kit was complete. Thirty-nine (91%) families had received training in administration of emergency intramuscular hydrocortisone, but two reported training had been several years prior and four (9%) reported no training.

Thirty-four (79%) families said that they were confident in administration of parenteral hydrocortisone. When asked, “can you think of a time when you could have given the injection but held back?” nine parents (21%) reported yes and 33 (79%) reported no. Reasons for not administering emergency medication included “being unsure if the child was unwell enough to warrant it” and “lack of confidence”.

Nineteen (44%) patients requested a refresher training course in clinic, 13 (30%) wanted a group refresher and 26 (60%) indicated that a video/booklet would be the most helpful way to improve confidence in this area.

## 4 Discussion

### 4.1 Adrenal Death

Incidence of AD in children and young people is rarely reported in the literature and few studies have tried to calculate this figure, preferring to describe causes of AD (13). The list of 107 comparison patients provided by the coding department was known to not represent all AI patients and thus could not be used to determine a definite incidence. The estimated incidence of one death per year and one death per 300 patient years is comparable with 1 AD per 205 patient years reported by Bensing et al. in children with primary adrenocortical insufficiency (9). These figures contrast with an incidence of 1 death per 1046 patient years in children with Type 1 Diabetes Mellitus (14), a figure already twice as high as that for children without a chronic condition. These figures highlight the seriousness of AI and the absolute importance that all healthcare providers are aware of the life-threatening nature of this condition. It also highlights the importance of recognising symptomatic adrenal insufficiency and administering treatment doses of hydrocortisone before progression to AC and AD.

No difference was found in IMD score between those with and without AD. It may be that deprivation does not have the effect on mortality that might be expected in this group or may simply reflect the limited use of a measure of deprivation based solely on postcode.

Our study was limited by the absence of information derived from relatives and families of the deceased and the definitive ascertainment of AC as a cause for AD is difficult and open to interpretation.

### 4.2 Emergency Department Attendances

Data from our analysis show that parent reported symptoms at home differ from those reported in medical notes: most parents in our survey reported a degree of reduced consciousness (70%) contrasting with only 11.8-14% in reports from healthcare staff (4, 12). This may represent the higher sensitivity of parents to their child’s normal activity level but could be secondary to pre-hospital parenteral hydrocortisone improving conscious level by the time of arrival at hospital in other studies.

The prevalence of pyrexia in more than half of patients with AC is similar in our study compared with those performed in hospital in other studies (72% (4)). The high number of AC episodes accompanied by pyrexia highlights the importance of educating parents about infection as a potential precipitant of adrenal crisis.

Parent education and advice to increase hydrocortisone dose with intercurrent illnesses is a vital part of the management of AI. All patients with AI in our regional centre are provided with face-to-face training at diagnosis by an endocrine nurse specialist and are signposted to a video developed in-house [youtu.be/Tx1yBcm5H0k], an App (MyCortisol) and website [addisonsdisease.org.uk/]. In our study, instructions to increase oral hydrocortisone dose were followed as per parent/patient education in all cases.

However, intramuscular hydrocortisone, also part of the education and training package for patients with AI, was not administered in the majority. This suggests that parents are aware of the importance of increasing hydrocortisone doses but reluctant to administer medication that must be injected. Interestingly, this reluctance to inject medicine was not identified in our follow up survey with 79% of parents reporting confidence in parenteral administration and only 21% reporting having missed an opportunity. While this follow up survey was conducted at a separate time, recruitment was from the same clinic and thus the patient population is likely reflective of the initial survey population and may even include some of the same patients. This contrast between perceived readiness to inject parenteral hydrocortisone and actual performance of this task when required is of concern and may suggest an inadequacy of training at diagnosis.

It is possible that the length of time from training at diagnosis to AC meant that training was no longer fresh and not utilised at the time of greatest clinical need. It is clear that existing methodology has low penetrance and that novel and more immersive learning/practical tools such as simulation models and virtual reality software may be necessary.

We hypothesised that AC might be more frequent in post codes in the UK associated with a lower level of affluence, considering the association between morbidity and deprivation in public health statistics (15) but our findings did not support this. This is likely for the same reasons described above for AD in that deprivation may not have the effect on morbidity that might be expected in this group or may simply reflect the limited use of a measure of deprivation based solely on postcode.

Our study is limited by the poor response rate of only 52% to the initial questionnaire and this does subject the findings to possible bias even though demographic comparisons between groups revealed no differences. However, the strength of the study lies in its focus on patients and families and their perceptions of both acute illness and its emergency treatment.

## Conclusion

During intercurrent illness episodes, parents reliably increased oral hydrocortisone doses but rarely administered emergency intramuscular hydrocortisone. Our study highlights the need for improved patient/parent education and training to reduce the risk of serious consequences in a choice of usable formats including alternative strategies. We did not identify any demographic risk factors for either AC or AD, but this requires further exploration in larger cohorts.

## Data Availability Statement

The raw data supporting the conclusions of this article will be made available by the authors, without undue reservation.

## Ethics Statement

Ethical review and approval were not required for the study on human participants in accordance with the local legislation and institutional requirements. Written informed consent to participate in this study was provided by the participants’ legal guardian/next of kin.

## Author Contributions

CW collected additional data, performed all statistical analysis, and drafted the manuscript. AV collected the all the initial data including questionnaires in Part B and approved the final manuscript. IB helped design the overall study, led the review of postmortem reports in Part A, provided a second review of statistical analysis, provided expert input, and reviewed and approved the final manuscript. WL performed the questionnaire and interviews in Part C and approved the final manuscript. JJ facilitated the collection of data in Part A and B and approved the final manuscript. HS facilitated the collection of data in Part A and B and approved the final manuscript. NK facilitated the collection of data in Part A and B and approved the final manuscript. SB helped design the overall study, contributed to the collection of data on pathology reports of young people, and provided expert input as well as approving the final manuscript. PC helped design the overall study, provided expert input, and reviewed and approved the final manuscript. All authors contributed to the article and approved the submitted version.

## Conflict of Interest

The authors declare that the research was conducted in the absence of any commercial or financial relationships that could be construed as a potential conflict of interest.

## Publisher’s Note

All claims expressed in this article are solely those of the authors and do not necessarily represent those of their affiliated organizations, or those of the publisher, the editors and the reviewers. Any product that may be evaluated in this article, or claim that may be made by its manufacturer, is not guaranteed or endorsed by the publisher.
